# Improving Emergency Department radiology transportation time: a successful implementation of lean methodology

**DOI:** 10.1186/s12913-017-2488-5

**Published:** 2017-09-05

**Authors:** Eveline A. Hitti, Ghada R. El-Eid, Hani Tamim, Rana Saleh, Miriam Saliba, Lena Naffaa

**Affiliations:** 1 Department of Emergency Medicine, American University of Beirut Medical Center, Beirut, Lebanon; 2 American University of Beirut Medical Center, Beirut, Lebanon

## Abstract

**Background:**

Emergency Department overcrowding has become a global problem and a growing safety and quality concern. Radiology and laboratory turnaround time, ED boarding and increased ED visits are some of the factors that contribute to ED overcrowding. Lean methods have been used in the ED to address multiple flow challenges from improving door-to-doctor time to reducing length of stay. The objective of this study is to determine the effectiveness of using Lean management methods on improving Emergency Department transportation times for plain radiography.

**Methods:**

We performed a before and after study at an academic urban Emergency Department with 49,000 annual visits after implementing a Lean driven intervention. The primary outcome was mean radiology transportation turnaround time (TAT). Secondary outcomes included overall study turnaround time from order processing to preliminary report time as well as ED length of stay. All ED patients undergoing plain radiography 6 months pre-intervention were compared to all ED patients undergoing plain radiography 6 months post-intervention after a 1 month washout period.

**Results:**

Post intervention there was a statistically significant decrease in the mean transportation TAT (mean ± SD: 9.87 min ± 15.05 versus 22.89 min ± 22.05, respectively, *p*-value <0.0001). In addition, it was found that 71.6% of patients in the post-intervention had transportation TAT ≤ 10 min, as compared to 32.3% in the pre-intervention period, *p*-value <0.0001, with narrower interquartile ranges in the post-intervention period. Similarly, the “study processing to preliminary report time” and the length of stay were lower in the post-intervention as compared to the pre-intervention, (52.50 min ± 35.43 versus 54.04 min ± 34.72, *p*-value = 0.02 and 3.65 h ± 5.17 versus 4.57 h ± 10.43, *p* < 0.0001, respectively), in spite of an increase in the time it took to elease a preliminary report in the post-intervention period.

**Conclusion:**

Using Lean change management techniques can be effective in reducing transportation time to plain radiography in the Emergency Department as well as improving process reliability.

## Background

The Emergency Department (ED) overcrowding has become a global problem and a growing safety and quality concern, with many studies demonstrating the negative impact of overcrowding on patient satisfaction as well as key quality metrics including total ED length of stay, percentage of patients left without being seen, and patient safety [1]. The main factors thought to be driving the overcrowding epidemic include rising ED visits, an aging population and relatively few inpatient beds [1]. In addition, the increased usage of imaging in the ED and the respective turnaround time for results has also been implicated [2, 3]. The 2012 US National Hospital Ambulatory Survey found that 46.8% of ED patients underwent any imaging in the ED compared to 40.7% in 2002 [4]. Furthermore, studies have demonstrated that patients who undergo imaging have longer ED visits, with one study reporting 4.4 times increased likelihood of remaining in the ED over 4 h if imaged [5, 6].

Much of the focus on improving ED throughput has been on addressing the ED boarder problem and inpatient bed availability; ED boarders are patients who end up waiting in the ED after a decision to admit has been made because of lack of inpatient beds. Although ED boarding is a serious problem with established safety concerns and one that places a heavy burden on ED resources, the solution is often tied to inpatient bed availability and hospital throughput, both of which involve many stakeholders well beyond the ED [7–10]. Designing processes to reduce turnaround time of ED studies can help improve ED flow, especially for low acuity patients, −many of whom require radiographic imaging and end up being discharged from the ED and are therefore not part of the inpatient bed bottleneck.

The success of Lean, the Toyota Production System management methodology, in manufacturing has led to its integration in healthcare systems and particularly in the ED. A report published in March 2009 by the American Society of Quality showed that out of the total surveyed U.S hospitals, 53% have reported using lean techniques, with emergency departments having 60% of lean deployment [11]. Two of the key principles of Lean methodology are eliminating unnecessary waste while maximizing value to the customer and ensuring continuous flow of work with minimal delays [12–16]. Lean methods have been used in the ED to address multiple flow challenges from improving door-to-doctor time to reducing length of stay [11, 12]. In radiology, Lean methodology has been used to improve multiple metrics including lowering waiting times for outpatient studies, patient waiting time for study completion in the radiology department, and report turnaround times [17].

To our knowledge, little has been done on the transportation part of the process of radiology turnaround time in the ED. This study aimed to assess the impact of a Lean-driven intervention on transportation turnaround time for plain radiography in an ED at a tertiary care center in Lebanon.

## Methods

### Study setting and design

The study was conducted at the ED of the American University of Beirut Medical Center (AUBMC) which is the largest tertiary care center in Lebanon. The ED volume at the time of the study was around 49,000 patients per year. Patients are triaged to three sections of the ED (High acuity, Low Acuity and Pediatrics) based on Emergency Severity Index (ESI) scoring. Admission rate in our setting is only 17% with the majority of patients being discharged home. The ED at AUBMC has a general radiography machine within its premises with a team of ED orderlies who, amongst other responsibilities, transport patients from all three sections to the radiography suite. Of all diagnostics studies performed in our ED, 68% are plain radiographs, while 29% and 3% are CT and ultrasonography respectively. Moreover, 30% of patients who present to the ED undergo plain radiographic imaging; in the low acuity section of our ED, this number goes up to 45%. All studies are read by radiology residents who release a preliminary read through the Picture Archiving and Communication System (PACS) with a subsequent final attending read usually reported within 48 h. Given the long turnaround for final read, decisions in the ED are based on either the ED attending impression or the radiology resident reported preliminary read.

The study was a pre- and post-intervention design comparing outcomes in both time periods. The 6 months pre-intervention period ranged between October 17, 2012 and April 17, 2013. After a 1 month washout period, the 6 months post-intervention period ranged between May 19, 2013 and November 19, 2013.

The study was deemed exempt from human subject research by the Institutional Review Board of AUBMC and conforms to the Declaration of Helsinki provisions.

### Theoretical model of the problem and LEAN intervention

Lean methodology focuses on reducing unnecessary waste within a system or a process to achieve smooth workflow. Key principles of lean include maximizing value to end users by removing waste, achieving a smooth workflow with little or no delays (heijunka), empowering frontline staff to make improvements, solving problems at the source, and continuous cycles of improvement initiatives. Common tools employed in Lean are Value Steam Mapping which involves identifying process steps and segregating value-added verus non-value added (waste) activities; kaizen, which involves assembling teams from across levels bringing together front-liners with management and other stakeholders to help achieve continuous process improvements; and kanban, which is the use of informatics to develop pull systems [11].

The ED at AUBMC has a process improvement committee that includes the ED chairperson, the ED medical director, two nurses including the ED nurse manager, case management, clerks, registration staff, as well as a hospital administrator with expertise in change management. This Kaizen team, after analyzing the trends in patient comments and complaints, decided to address ED length of stay with specific focus on turnaround time of radiology studies. Following the theory of constraints for tackling bottlenecks that calls for a focus on the most important limiting factor to a process [18], the team decided that focusing on the turnaround time for plain radiography is key to improving ED throughput especially for low acuity patients. The clinical department administrator of the radiology department was thus invited to be part of the improvement. The team first divided the overall study turnaround time (from study order entry to preliminary report release time) into four main steps:

1. “Order entry to order processing time”: the time the medical staff places an order through the computerized order entry system to when the request is financially processed by the cashier, after which the order status changes to pending in the order system
2. Transportation turnaround time (transportation TAT): the time the order is processed by the cashier to the time the radiographer initiates the study as registered through the PACS system.
3. “Study initiation to study completion time”: time the radiographer initiates the study as registered by the PACS system to the time the study is completed.
4. Preliminary report turnaround time (preliminary report TAT): the time the study is completed to the time the radiology resident releases a preliminary report on the PACS system.

**Fig. 1:**
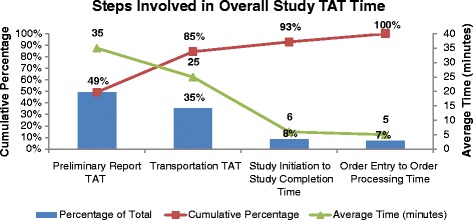
Pareto diagram of overall study turnaround time

Time motion mapping of the process showed turnaround times in the following descending order: preliminary report TAT, transportation TAT, “study initiation to study completion time”, and “order entry to processing time” (Fig. 1). Even though the greatest wait was in preliminary report TAT, the team decided to focus on the transportation TAT, as they felt the preliminary report TAT would require changing the process of the radiologists reporting the preliminary reads which was beyond the scope of the ED committee.

**Fig. 2:**
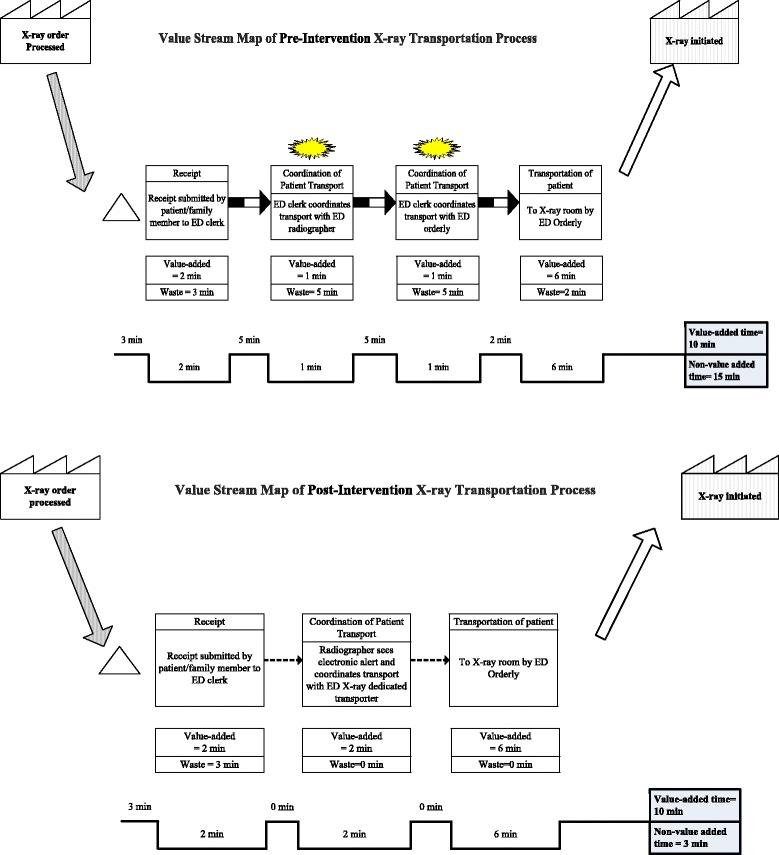
Value Stream Map of the pre- and post-intervention transportation process

Figure 2 presents the Value Stream Map of the transportation process which was completed to help identify value-added versus non-value added activities across all process steps. The original process was highly clerk-dependent, requiring the clerk to initiate separate pages to the ED orderly and the radiographer to coordinate availability of each to take the patient to the x-ray room, after receiving the imaging request. The ED orderly are ancillary nursing support staff who have multiple responsibilities including transporting patients from triage to the different ED sections, store keeping and transportation of patients to inpatient beds. Coordinating ED orderly availability with that of radiographer availability led to a chain of back and forth phone calls and repeat paging messages that the clerk had to follow up on, amongst their own multitude of tasks. In addition, lack of visibility of pending studies to the radiographer led to dependence on ED clerk for sporadic information on pending requests with little ability to manage workload and pace. The revised process included two main changes: firstly, a dashboard of all pending requests that included patient name, medical record number, location and type of study was created for the radiographer; secondly, during the day from 8 am-11 pm, when ED and study volumes are highest, a dedicated transporter was assigned to the radiography room alongside the radiographer with no other responsibilities beyond patient transport back and forth to the radiology room. The clerk was thus entirely removed from the process, replaced by the dashboard for study notification and a dedicated transporter for direct in-person communication and coordination of work.

### Sampling

All plain radiographs ordered and completed for patients seen in the ED during the study time periods were included. We excluded all radiographs done between midnight and 8:00 am, during which time there was no dedicated radiography transporter. In addition, all radiographs on patients who left against medical advice or were transferred to other hospitals were excluded from the analyses. This was done as the length of stay for such patients would be affected by factors beyond the ED or institutional processes.

### Outcome measure

The main outcome measured was the mean transportation TAT. Although our intervention did not target parts of the process beyond the transportation time, specifically preliminary report TAT or “study processing to preliminary report time”, these were important to assess because physician decision making and ultimate length of stay are dependent on preliminary report availability. We also looked at length of stay of patients in the ED pre- and post-intervention with specific focus on ESI 4 and 5 patients whose length of stay is expected to be affected by overall study TAT.

### Data collection

Order processing time was retrieved from our billing system while the study initiation time was retrieved from our PACS system. The preliminary report time was retrieved from the PACS system and included the time the first preliminary result was released by the radiology resident for viewing.

Patient characteristics were collected from our administrative database and included age, gender, guarantor coverage, ESI, imaging required and disposition. This database was also used to pull ED characteristic that could potentially impact transportation time including: volume of ED visits per day, shift type (day: 8 am-4 pm; evening: 4 pm-midnight), and day of the week (weekend: Saturday and Sunday; weekday: Monday to Friday, inclusive).

### Data analysis

Data was entered and managed using the Statistical Package for Social Sciences (SPSS), version 22. Descriptive analyses were carried out by reporting the mean and standard deviation (± SD) for continuous variables, whereas number and percent were used for categorical ones. Association between the pre- and post- intervention and different categorical variables was done using the Pearson chi-square test. On the other hand, Student’s t-test was used to compare continuous variables. Moreover, boxplots were constructed for the pre- and post-intervention for the Transportation TAT by the ESI level, where median and interquartile range (IQR) were reported. To account for confounding variables, multivariate analyses were carried out, mainly logistic regression for categorical variables, or linear regression for continuous ones. Results of the regression analyses were reported as odds ratio (OR) for categorical outcomes, or coefficient estimates for continuous ones, along with the corresponding 95% confidence interval (CI). A *p*-value less than 0.05 was used to denote statistical significance.

## Results

A total of 6186 and 4879 radiographs were included in each of the 6 months pre- and post-intervention periods, respectively (Table 1). Regarding patient-related metrics, patients who underwent radiographic imaging in the post-intervention period were older as compared to the pre-intervention period (43.4 years ±25.9 and 41.7 years ±26.2, respectively), *p*-value = 0.001. Moreover, more patients who underwent radiography in the post-intervention period presented with intermediate complexity (ESI 3) than the pre-intervention phase (69.9% versus 63.6%, respectively, *p*-value <0.0001). In addition, in the post-intervention period, fewer patients underwent chest x-rays compared to the pre-intervention period (45.3% versus 49.2%, respectively, *p*-value = 0.002). Furthermore, in the post-intervention period, more patients who required imaging presented during the day shift as compared to the pre-intervention period (51.8% versus 47.3%, respectively, *p*-value <0.0001). There were no differences in gender, guarantor, disposition, or weekend / weekday presentations for patients undergoing imaging between the two time periods. The ED was however busier in the post-intervention period, with 137 ± 17 compared with 133 ± 17 average visits per day (*p*-value <0.0001) (Table 1).

**Table 1:** Baseline characteristics of patients in the pre- and post-intervention periods

		Intervention		
Variables		Pre *n* = 6186	Post *n* = 4879	*P*-value
Age	Mean (±SD)	41.7 ± 26.2	43.4 ± 25.9	0.001
Gender	Female	2803 (45.3%)	2176 (44.6%)	0.45
Guarantor	Self-Pay	1220 (19.7%)	1005 (20.6%)	0.49
	Private insurance	4866 (78.7%)	3792 (77.7%)	
	Other	100 (1.6%)	82 (1.7%)	
ESI	1	45 (0.7%)	21 (0.4%)	<0.0001
	2	438 (7.1%)	230 (4.7%)	
	3	3936 (63.6%)	3411 (69.9%)	
	4	1704 (27.5%)	1189 (24.4%)	
	5	63 (1.0%)	28 (0.6%)	
RAD category	Chest xray	3041 (49.2%)	2212 (45.3%)	0.002
	Abdomen xray	164 (2.7%)	128 (2.6%)	
	Extremities xray	2540 (41.1%)	2168 (44.4%)	
	Head and Neck xray	60 (1.0%)	58 (1.2%)	
	Spine xray	381 (6.2%)	313 (6.4%)	
Disposition	Admitted	1582 (25.6%)	1304 (26.7%)	0.17
	Discharged	4604 (74.4%)	3575 (73.3%)	
Day of the week	Weekday	4380 (70.8%)	3522 (72.2%)	0.11
	Weekend	1806 (29.2%)	1357 (27.8%)	
Radiographer Shift	Day	2924 (47.3%)	2528 (51.8%)	<0.0001
	Evening	3262 (52.7%)	2351 (48.2%)	
ED characteristics (volume per day)	Mean (±SD)	133 ± 17	137 ± 17	<0.0001

Table 2 presents the association between the intervention and the outcomes considered in this study. Transportation TAT was found to decrease in the post-intervention period as compared to the pre-intervention (mean ± SD: 9.87 ± 15.05 and 22.89 ± 22.05, respectively, *p*-value <0.0001). In addition, it was found that 71.6% of patients in the post-intervention had transportation TAT ≤ 10 min, as compared to 32.3% in the pre-intervention period, *p*-value <0.0001. Similarly, the “study processing to preliminary report time” and the length of stay were lower in the post-intervention as compared to the pre-intervention, *p*-values = 0.02 and <0.0001, respectively. On the other hand, the preliminary report TAT was found to increase in the post-intervention period (*p*-value <0.0001).

**Table 2:** Comparison of outcomes between pre- and post-intervention periods

		Intervention		
Variables		Pre *n* = 6186	Post *n* = 4879	*P*-value
Intervention related				
Transportation TAT (min)	≤10	1998 (32.3%)	3494 (71.6%)	<0.0001
	Mean (±SD)	22.89 ± 22.05	9.87 ± 15.05	<0.0001
Others				
Preliminary Report TAT (min)	Mean (±SD)	31.16 ± 26.87	42.63 ± 33.02	<0.0001
Study processing to preliminary report time (min)	Mean (±SD)	54.04 ± 34.72	52.50 ± 35.43	0.02
Length of stay (hrs)	Mean (±SD)	4.57 ± 10.43	3.65 ± 5.17	<0.0001

After adjustment for the potentially confounding variables, the results of the multivariate analyses for the association between the intervention and the outcomes are presented in Table 3. The adjusted beta coefficient for the transportation TAT was found to be −13.00 (95% CI: -13.70; −12.31) for the post-intervention as compared to the pre-intervention. Similar to the unadjusted analyses, both the “study processing to preliminary report time” and the length of stay dropped in the post-intervention period, whereas the preliminary TAT increased.

**Table 3:** Multivariate analyses for the association between pre- and post-intervention and outcomes

		Measure of association	*P*-value
Intervention related			
Transportation TAT	> 10 vs ≤ 10	aOR (95% CI)	<0.0001
		0.16 (0.15; 0.18)	
	min	Adjusted Beta (95% CI)	<0.0001
		−13.00 (−13.70; −12.31)	
Others			
		Beta (95% CI)	
Preliminary Report TAT	min	10.69 (9.59; 11.78)	<0.0001
Study processing to preliminary report time	min	−2.31 (−3.58; −1.04)	<0.0001
Length of stay	hrs	−1.02 (−1.33; −0.72)	<0.0001

Variables entered in the model are: Radiology (reference: GR & XR chest), Day of the week (reference: Weekday), Volume per day, Radiographer shift (reference: Day), Age (per 10 units increase), Gender (reference: Male), Guarrant (reference: Self paying patients), and ESI. aOR: adjusted odds ratio

Finally, Table 4 presents the stratified analyses of the association between the intervention and the outcomes by different patient and ED characteristics subgroups. The intervention was associated with a bigger drop in transportation TAT among high acuity patients (ESI ≤3) as compared to the low acuity (ESI >3), (adjusted beta: -15.17, 95% CI: -16.07; −14.27 and −7.63, 95% CI: -8.41; −6.84, respectively), *p*-value for interaction <0.0001. Similarly, the intervention impacted the transportation TAT for admitted patients to a greater extent as compared to those who were discharged, *p*-value interaction <0.0001. In addition, the intervention was more effective in the evening shifts and weekdays compared to the day shifts and weekends. As for the length of stay, it was found that the intervention was associated with a higher drop among high acuity patients, admitted patients, weekday shifts and higher volume per day shifts.

**Table 4:** Multivariate analyses for the association between pre- and post-intervention outcomes in different patient and ED characteristic subgroups

	Measure of association	*P*-value	*P*-value for interaction
Transportation (min)			
	Adjusted Beta (95% CI)		
ESI			
≤ 3 (*n* = 8080)	−15.17 (−16.07; −14.27)	<0.0001	<0.0001
> 3 (*n* = 2983)	−7.63 (−8.41; −6.84)	<0.0001	
Disposition			
Admitted (*n* = 2886)	−19.61 (−21.46; −17.76)	<0.0001	<0.0001
Discharged (*n* = 8177)	−10.65 (−11.31; −9.99)	<0.0001	
Radiographer Shift			
Day (*n* = 5451)	−11.98 (−12.91; −11.05)	<0.0001	0.002
Evening (*n* = 5612)	−14.01 (−15.05; −12.98)	<0.0001	
Day of the week			
Weekday (*n* = 7900)	−13.49 (−14.32; −12.67)	<0.0001	0.03
Weekend (*n* = 3163)	−11.58 (−12.86; −10.29)	<0.0001	
Volume per day			
≤ 134 (*n* = 5679)	−12.79 (−13.78; −11.80)	<0.0001	0.41
> 134 (*n* = 5384)	−13.26 (−14.23; −12.28)	<0.0001	
Length of stay (hrs)			
ESI			
≤ 3 (*n* = 8080)	−1.42 (−1.84; −1.00)	<0.0001	<0.0001
> 3 (*n* = 2984)	−0.16 (−0.29; −0.04)	0.01	
Disposition			
Admitted (*n* = 2886)	−3.91 (−5.01; −2.81)	<0.0001	<0.0001
Discharged (*n* = 8178)	−0.05 (−0.13; 0.02)	0.17	
Radiographer Shift			
Day (*n* = 5451)	−1.26 (−1.70; −0.83)	<0.0001	0.15
Evening (*n* = 5613)	−0.81 (−1.24; −0.37)	<0.0001	
Day of the week			
Weekday (*n* = 7901)	−1.33 (−1.72; −0.93)	<0.0001	0.004
Weekend (*n* = 3163)	−0.26 (−0.66; 0.14)	0.20	
Volume per day			
≤ 134 (*n* = 5679)	−0.58 (−0.94; −0.23)	0.001	0.01
> 134 (*n* = 5385)	−1.41 (−1.91; −0.91)	<0.0001	

**Fig. 3:**
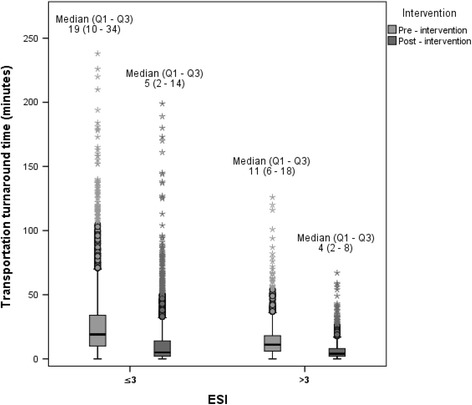
Boxplot of the transportation turnaround time pre- and post-intervention, by ESI

Figure 3 presents boxplots of the transportation TAT for the two intervention periods stratified by high and low acuity patients. There was a drop in median transportation TAT in the post- intervention period as compared to the pre-intervention, as well as having a narrower interquartile range in the post-intervention. This finding was found in both the low and high acuity patients, where it was more profound for the latter.

## Discussion

In our setting, Lean methodology was effective in reducing transportation time of patients to plain radiography in the ED. The process redesign focused on allowing the radiographer to “pull” patients to imaging by improving his/her visibility of pending orders and removing the clerk from the process by assigning one of the ED orderlies to the radiography suite during peak hours. Even though the preliminary report turnaround time increased in our study period, the significant improvements in transportation time reduced overall study turnaround time from order processing to preliminary report time.

Recent studies targeting turnaround times of radiological imaging in the ED have focused on TAT of imaging reads, from study completion to issue of radiology report [3, 19–22]. Improvements in this component of overall TAT for radiology reads usually require increased personnel, investment in new technology and addressing physician behavior and practice which is often challenging and beyond the direct scope of ED management team [22]. Our study is the first to address the transportation process, specifically the order processing to study completion step of the process in the ED, which we believe was lower hanging fruit and one that involved more process design and system issues than human behavior. Not only did the transportation TAT drop post-intervention from 22.9 to 9.9 min, but the reliability of the process improved with the majority (71.6%) of patients being transported within 10 min compared to only 32.3% in the pre-intervention period. The narrower interquartile ranges in the post-intervention period also reflect a more reliable process.

While there has been much focus on improving the “front-end” and “back-end” problem of ED throughput by tackling “Door to Doctor time” and bottlenecks to the inpatient bed availability [23], focusing on ways to reduce the length of stay by identifying bottlenecks within the patient visit can also improve flow, especially for patients who will ultimately be discharged from the ED. While patients’ length of stay decreased in the post-intervention phase, it was however more prominent for admitted than discharged patients. This could be explained by the higher impact of the intervention on admitted patients, which may have been related to the proximity of the radiology suite to the high acuity section of the ED compared to the low acuity section. In addition, there was an increase in the preliminary report TAT post-intervention, likely related to internal operational changes within the radiology department, which may have dampened the overall impact of our intervention on study turnaround time and thus length of stay. Preliminary report TAT in our institution is dependent on radiology resident reading of ED images and entry of a preliminary report in the PACS system. During our study period, the Radiology Department underwent a change in leadership, including a new Chairperson and residency program director, that may have impacted prioritization of ED image reading in the residents’ daily work. Inclusion of radiology department physician champion within our kaizen team may have prevented these observed delays by improving alignment of priorities between departments. Furthermore, the reduction of length of stay from 4.57 to 3.65 h post-intervention is well beyond the 13 min improvement in transportation time. This could be explained by operational changes that were directed at reducing the door to doctor time in our ED during the same time period. These included demand-capacity matching of physicians and nurses to patient volumes, the introduction of bedside registration, team distribution of patients within sections and immediate bedding of high acuity patients [12]. Although all these interventions likely reduced overall length of stay by reducing the door to doctor time, they were unlikely to impact transportation turnaround time as they targeted a step preceding the diagnostic ordering process.

While The Joint Commission has recently started urging hospitals to use specific change methodology tools including Lean and Six Sigma to develop more reliable processes, some of the evidence for use of Lean in the healthcare setting has been criticized for weak methodology [24]. Reviews of the literature on effectiveness of Lean methodology specifically in the ED or radiology setting, though generally positive, identify methodological concerns related to sample size of existing studies or failure to demonstrate sustained impact [11, 17]. Our study includes a large pre- and post-intervention analysis of 11,065 radiographic images and included transportation times up to 7 months post intervention with sustained improvements.

The key Lean principles we relied on for our intervention were eliminating unnecessary waste related to the clerk paging the orderly/radiographer in the original process and achieving smooth flow (*heijunka*) by removing the clerk from the process. The three main Lean tools our team found useful for this project were: firstly, establishing a *kaizen* team that included and engaged all key stakeholders; secondly, value stream mapping which assisted everyone in identifying the sources of waste; and finally, creating information systems that allowed the radiographer to know when the patient was ready to be “pulled” to the radiography suite (*kanban*). Furthermore, ultimate reassignment of orderly roles with dedication of one of the orderlies to the radiography suite where the team believed one of the largest internal bottlenecks existed, was key to improving coordination of the team involved in the transportation process.

### Limitations

Due to the pre-post intervention design with retrospective data review, not all potentially confounding variables that might have biased the results were assessed, although we captured the major factors and included them in the multivariate analyses. In addition, our post intervention sample is smaller; this is likely due to seasonal variations including increased volumes related to the flu season. Moreover, we only assessed the process and efficiency metrics but did not measure quality of the preliminary reads or discrepancy read rates during the study period as we do not believe our intervention could have impacted these metrics. Furthermore, patient satisfaction was not assessed, which could be addressed in future research.

## Conclusion

In summary, Lean methodology was implemented successfully in our setting and improved ED transportation TAT for plain radiography as well as the process reliability. The Kaizen team focused on a process step within the ED control and one that was felt to be a main constraint for throughput of low acuity/discharged patients whose length of stay is not impacted by the inpatient bed bottleneck. Although the intervention was successful, ultimate impact on length of stay was dampened by the increase in preliminary report TAT during the post-intervention period which was beyond the control of the ED team. Expanding the Kaizen team to include radiology physician members could have improved the impact of our overall study TAT by preventing delays related to preliminary reporting. Value focus and use of information system to allow the radiographer to pull patients back for imaging were key components of the success of this intervention. Other Emergency Department managers can easily adopt lean tools specifically kaizen team, value stream mapping, and kanban system to improve throughput related metrics in their specific settings.
